# Medicolegal Cases in Bariatric Surgery in the United Kingdom

**DOI:** 10.1007/s13679-023-00508-1

**Published:** 2023-06-02

**Authors:** Matyas Fehervari, Michael G. Fadel, Marcus Reddy, Omar A. Khan

**Affiliations:** 1 Department of Bariatric Surgery, St George’s Hospital, London, UK; 2 Department of Surgery and Cancer, Imperial College London, London, UK; 3 Population Sciences Department, St George’s University of London, London, UK

**Keywords:** Breach of duty of care, Negligence, Litigation, Emergency surgery

## Abstract

**Purpose of review:**

To evaluate the current state of bariatric medicolegal activity and explore the reasons of litigation in bariatric surgery. The underlying legal principles in bariatric medicolegal cases and most frequent pitfalls will also be discussed.

**Recent findings:**

There is a growing number of litigations in bariatric surgery, particularly relating to complications and long waiting lists for bariatric surgery within the public-funded health systems. The main issues are related to consent, lack of follow-up, delayed identification of complications and lack of appropriate emergency management of complications, involving bariatric surgeons, clinicians, general practitioners and multidisciplinary team members. Appropriate multidisciplinary involvement pre- and postoperatively and robust follow-up protocols can help to mitigate the risks.

**Summary:**

Bariatric surgery requires a unique paradigm with a multidisciplinary approach both pre- and postoperatively to improve the long-term functional outcomes of patients. There is a rising incidence of medicolegal claims following bariatric surgery. The underlying reasons for this are multifactorial including an increase in the volume of surgery, high patient expectations, the incidence of long-term postoperative complications and the requirement of long-term follow-up.

## Introduction

Obesity is associated with severe morbidity such as hypertension, dyslipidaemia, diabetes mellitus, congestive heart failure and cancer, and has been shown to reduce life expectancy by 9.8 years compared to normal body mass index (BMI [weight/height^2^]) [1]. Bariatric surgery remains the most effective method in permanently reducing weight in patients with obesity who have not responded to lifestyle modifications or pharmacotherapy [2•, 3]. It can enhance quality of life and improve obesity-related comorbidities leading to a reduction in long-term mortality [4].

Over the last decade, there has been a steady decrease in complication rates and perioperative mortality associated with bariatric surgery in the United Kingdom (UK), Europe and the United States of America (USA) [5–7]. For example, the 2020 UK National Bariatric Surgical Register reported a postoperative mortality rate of 0.04% and complication rate of 2.4% for bariatric surgical procedures, equally favourable outcomes were also demonstrated in Europe and the USA [8–10]. However, the increase in frequency of bariatric surgery has been accompanied by an increase in litigation involving patients and practitioners, as well as rising medical insurance premiums both in the UK, Europe and USA [8, 11]. Bariatric surgery is now reported as one of the highest litigation risks by surgeons in the USA with them being defendants in 92% of the cases [12••, 13•]. In terms of total number of cases there were nine claims between 1990 and 1999 (0.9/year), 194 claims between 2000 and 2009 (19.4/year) and 175 claims between 2006 and 2014 (21.86 year) in the USA [14••, 15]. There is a centralised registry for closed claims in the USA [15]. In contrast, in the UK, there were 7 reported cases between 2003 and 2013 (0.7/year) [16••]. In Europe, there was an increase of the proportion of bariatric surgery cases within medico-legal litigations [17]. However it is difficult to be certain about the total number of litigations in Europe and the UK due to the lack of publicly available registry and it is not mandatory to report cases to the National Litigation Authorities. Nevertheless, it is useful for bariatric surgeons to recognise the types of cases which can lead to potential malpractice claims and to be develop strategies that can help avoid pitfalls in order to provide optimal care for their patients and their own practice.

This review article aims to evaluate the current state of bariatric medicolegal activity and explore the reasons for increasing litigation in bariatric surgery. We will also discuss the underlying legal principles in bariatric medicolegal cases and the common pitfalls a surgeon may encounter.

## Bariatric Surgery and Litigation

A rising global prevalence of obesity and an increase in the number of bariatric procedures has resulted in an overall increase in the number of potential litigations [8, 18, 19•]. This is highlighted by a retrospective analysis of claims associated with morbid obesity performed by Weber et al. [14] using the Physician Insurers Association of America database, between 1990–1999 and 2000–2009. Of the 575 claims identified, gastric bypass was the most frequent procedure and the number of morbid obesity claims increased from nine during the initial period to 249 in the subsequent period, which was deemed to be a result of a significant increase in the number of bariatric operations performed. Other common operations leading claims fallen in to the category of ‘operative procedures on the stomach’ according to the database.

Bariatric surgery can be technically challenging and should be performed in specialist units. In some countries, most commonly where healthcare is government-funded, like the UK or Canada, there are long waiting lists for bariatric surgery which has been impacted by the COVID-19 pandemic. There is limited centralised information on the exact waiting times; however, available resources suggest that the waiting times can be between 30 weeks and 5 years in these countries [20–24]. To avoid the long waiting lists in the public sector, some patients may choose to seek bariatric surgery in the independent sector often self-funded. As a consequence, these patients tend to have a lower threshold for making a complaint should something go wrong, particularly having paid for their operation. In summary, patient satisfaction, amongst others, is a factor that drives patients to seek litigation.

To minimise the costs of private healthcare, some patients may also seek to have their surgical procedures abroad and may experience varying outcomes and follow-up. This may result in a more complex litigation process if patients develop complications as they may seek redress against their local providers for denying them initial access to surgery.

Although obesity is associated with significant medical comorbidities, it is not considered to be immediately life-threatening [25•]. The mortality benefit is long-term and patients who choose to undergo surgery do so to improve their health and quality of life. As a result, there is poor tolerance to postoperative complications should they occur leading to patient dissatisfaction and litigation. In contrast to conventional surgery where a pathological abnormality is resected or repaired, in bariatric surgery normal anatomy is being deliberately (and often irreversibly) disrupted. This can lead to several late procedure-specific complications, such as gastric ulceration or internal herniation, or gastric band slippage or erosion, which are further potential sources of litigation.

Negligence related to bariatric surgery is often based on a failure to detect complications in a prompt manner as opposed to the complications themselves. Cottam et al. [26] reviewed the case files of 100 consecutive bariatric surgery lawsuits. The most common adverse events initiating litigation were anastomotic leaks followed by intra-abdominal abscess, bowel obstruction, major airway events, organ injury and pulmonary embolism. In terms of clinical outcomes, 32 patients had a documented intraoperative complication and 72 required subsequent surgery. Fifty-three patients died and 28 made a full recovery with the remainder having minor or major disability. Analysis by a medical malpractice lawyer revealed potential negligence in 28% of cases with delay in diagnosis of a complication or misinterpretation of vital signs being the most common cause. The majority of lawsuits involved surgeons with less than 1 year of experience in bariatric surgery.

Similarly, Brugera et al. [27] reviewed the case notes of 49 Spanish medicolegal bariatric surgery cases presented to the Professional Liability Department of the Catalonian Medical Colleges Council from 1992 to 2009. In 47% of the cases, the patients died, 21% made a complete recovery and the remainder had some residual impairment. Peritonitis due to anastomotic leaks and respiratory complications were the two most common causes of death. Malpractice was considered to have occurred in 20% of cases and in 6% of cases the surgeons were convicted in criminal courts of criminal negligence. In the UK, Ratnasingham et al. [16] analysed the claims data from the UK National Health Service (NHS) Litigation Authority and found seven claims for bariatric surgery, of which four were successful. This was secondary to inadequate consent, delay in treatment, retained instrument and inadequate duration of follow-up.

## Avoiding Medicolegal Pitfalls

Each country has its own legal terms. The legal proceedings in the UK and their relevance to bariatric surgery have been summarised in Table 1. Given the increasing incidence of medicolegal cases, it is important for clinicians involved in the care of bariatric patients to ensure that they are aware of possible medicolegal pitfalls. Some key areas which need to be addressed are described below.

**Table 1:** Legal test and proceedings in the UK and their relevance to bariatric surgery

**Legal Precedent**	**Description**	**Relevance to bariatric surgery**
Bolam test	Determine whether a doctor has breached their duty of care to a patient. The test states that a doctor is not negligent if they act in accordance with a responsible body of medical opinion.	Applies to all fields of surgery including bariatric surgery
Gillick competence	Describes the ability of a child under 16 years old to consent to medical treatment without the need for parental consent or knowledge. The test is based on the child’s ability to understand the nature and implications of the treatment.	May be relevant to bariatric surgery if the patient is under 16 years old
Jones ruling	Introduced the principle of “material risks” in medical consent and states that doctors must disclose risks to patients that a reasonable person in the patient’s position would consider to be significant.	Applies to all fields of surgery including bariatric surgery
Chester ruling	Introduced the principle of “best interests” in medical decision-making for patients who lack capacity. Decisions should be made in the patient’s best interests considering their wishes, feelings, beliefs and values.	Only relevant to Bariatric Surgery in an emergency setting where a patients’ capacity deteriorated following bariatric surgery

## Appropriate Consenting

The main elements of informed consent are that consent should be provided voluntarily by a patient with capacity after being provided information regarding the procedure including potential benefit, alternatives and risks. Legally, the duty of care to provide all the relevant information lies with the caregiver. This was evident in the UK case of Chester versus Afshar. In 2002, Miss Chester developed an uncommon complication of surgery, cauda equina syndrome, but was not informed of the possibility of this preoperatively. The judge found that there was a causal connection between the failure to inform and the complication that subsequently arose. Miss Chester would have sought further advice or alternatives if she had been adequately informed [28].

A significant portion of bariatric medicolegal complaints revolve around inadequate consent. In order to avoid such issues, it is essential that the consenting process is carefully documented with evidence that patients are provided with the options of both conservative treatment and surgery. With respect to surgical options, it is important that long-term significant and potentially life-threatening risks associated with surgery are emphasised. There should be evidence of full discussion with the patient prior to surgery on more than one occasion.

## Change in Operating Surgeon

Bariatric surgeons work with several surgical members ranging from consultants to fellows and more junior trainees. The patient may see one member of the team in clinic and meet a different person on the day of the procedure. When consenting, it is therefore very important to ensure that the patient is aware of this and that the surgery may be performed by a different member of the team. This specific issue has been the subject of litigation in the UK highlighted by an obstetric case of Jones versus Royal Devon and Exeter NHS Foundation Trust. In this case, the judge ruled that a change of the named surgeon (who had seen the patient preoperatively) to a surgical fellow without prior explicit consent constituted a breach of duty of care [29]. This ruling has clear implications not only for training but also for delivery of service where groups of surgeons usually add patients to a pooled waiting list.

## Consent in Children

Bariatric surgery in children and adolescents has been established as a safe and effective method for weight loss; however, it remains a divisive topic [30]. Consent in this group is further complicated as it is usually provided by a carer rather than the patient themselves, often resulting in the procedure being delayed until the patient attains adulthood and is able to consent themselves.

In the UK, a significant ruling in the 1985 created the concept of “Gillick competence” [31, 32]. This refers to a young person below the age of 16 years who has the intellectual and cognitive ability to make reasoned decisions about their own care—these patients are deemed as not requiring parental consent to undergo surgery. Thus far, there have been no reported cases of controversy relating to Gillick competence in bariatric surgery but with increasing childhood obesity rates, this is likely to be an important area for bariatric surgeons in the future.

## Emergency Presentation

Bariatric patients may present acutely many years following surgery with complications related to the initial operation. These patients pose a particular problem when they present to a hospital with limited bariatric experience; but, while such a facility will not be expected to provide expert bariatric care, it would be expected to be able to provide a diagnosis and discuss the management with an appropriate bariatric centre. Failure to do so, as discussed above, is a growing area for litigation.

## Medicolegal Cases

In order to highlight some of the issues above, four anonymised cases are described:

### Case Number 1

A 35-year-old female with a BMI 42 kg/m^2^ self-referred to a surgeon working in the private sector for consideration of bariatric surgery. The patient was offered a laparoscopic sleeve gastrectomy (LSG) operation following an outpatient appointment. The patient then recontacted the surgeon requesting a banded sleeve gastrectomy instead. The surgeon arranged for the patient to attend a second outpatient appointment at which point the surgeon agreed to perform the procedure for an additional cost. The patient was consented for a banded sleeve gastrectomy procedure and the procedure was completed uneventfully. Ten days postoperatively, the patient presented acutely unwell to the local emergency department. After initial assessment, she was immediately transferred to a bariatric centre and underwent a diagnostic laparoscopy where she was found to have a perforation in the midportion of the sleeve under the band. The band was removed and the perforation was repaired. Subsequently, the patient had an extended recovery and was eventually discharged from hospital after 2 months. On discharge, she was noted to have nerve neuropathy after her intensive treatment unit stay.

#### Medicolegal Analysis

The patient’s solicitors submitted a letter of claim alleging the following:

i. The patient had been inadequately counselled and consented for the procedure, and the surgeon performing the procedure was inadequately experienced in this operation.
ii. The development of the postoperative leak was evidence of a substandard surgical technique. If an appropriate surgical technique had been used, a postoperative leak would not have occurred.

The operating surgeon’s solicitors responded as follows:

iii. The patient had a specific consultation to discuss banded sleeve gastrectomy.
iv. Although, the surgeon performing the procedure was inadequately experienced in this operation, he was highly experienced in gastric band insertion and LSG, and had also performed primary banded gastric bypasses.
v. The surgeon’s operative technique was appropriate as evidenced by the fact that he had not had a leak from primary bariatric surgery in over 4 years.
vi. Postoperative leak is an accepted complication following standard performance of the sleeve gastrectomy.

#### Expert Review

Although the surgeon performing the procedure had the technical skills to perform the operation and had undertaken a specific consultation to discuss the banded sleeve gastrectomy, the fact that they had never performed a banded sleeve gastrectomy was material information which was not given to the patient. As such, under the ‘prudent patient’ test, the consent process could not be defended due to this omission. With regard to the materiality of the breach, the experts accepted that leaks were a recognised complication following sleeve gastrectomy, and that the operation itself (based on the documentation) appears to have been done in an appropriate fashion. However, if the surgeon stated to the patient that he had never performed a banded sleeve gastrectomy, it is likely that the patient would have opted to undergo a standard sleeve gastrectomy. Based on the fact that the perforation was noted to be at the site of the band, and the fact that the surgeon had (by his own admission) not had a leak from a primary sleeve gastrectomy in over 5 years, on the balance of probabilities it was felt that in these circumstances the claimant would have avoided a leak had she undergone a sleeve gastrectomy and therefore, liability should be conceded by the operating surgeon.

#### Learning Points

This case demonstrates the importance of disclosing all material facts to the patient as part of the consenting process. Although it was reasonable to offer the patient this operation, and there was no suggestion that there were any technical issues with the surgery, the absence of full disclosure of the surgeon’s experience with this particular operation invalidated the consent and as a consequence, the patient’s claim was successful.

### Case Number 2

A 42-year-old female with a BMI 46 kg/m^2^ self-referred to a surgeon working in the private sector for consideration of bariatric surgery. She consented for a laparoscopic Roux-en-Y gastric bypass which was performed uneventfully. Three months postoperatively, it was noted that the patient has excellent weight loss but reported ongoing nausea and abdominal pain. The patient’s symptoms persisted, and she underwent an upper gastrointestinal (GI) endoscopy and gastrograffin swallow which showed no abnormalities. One year following surgery, the patient was discharged from the care of her private surgeon as per her agreed package of care with instructions to contact her primary physician if she had any issues. The patient still had persistent malaise and nausea and saw her primary physician who referred her to the gastroenterology outpatient clinic for further investigations. In the clinic, she was found to have excellent weight loss with a BMI of 20 kg/m^2^. Routine blood tests revealed deranged liver function tests and a low albumin. An ultrasound showed a gallstone within a thin-walled gallbladder and a magnetic resonance cholangiopancreatography revealed no abnormalities. A percutaneous liver biopsy revealed non-alcoholic steatosis. Three months after her initial gastroenterology review, she was admitted as an emergency with peritonitis. At laparotomy, she had an internal hernial defect in Petersen’s space with gross dilatation and perforation of the blind end of the biliopancreatic limb consistent with a longstanding obstruction. The blind end of the biliopancreatic limb was resected; however, the patient had a prolonged period of sepsis and died 2 weeks postoperatively.

#### Medicolegal Analysis

The patient’s solicitors submitted a letter of claim alleging the following:

i. The presence of an internal hernia was a direct consequence of the negligent failure of the surgeon to close the mesenteric defects intraoperatively.
ii. The failure of the bariatric surgeon and gastroenterologists to diagnose the presence of an internal hernia of the biliopancreatic limb following surgery was a breach of duty of care.

#### Expert Review

Expert opinion was supportive of the decision not to close the mesenteric defects at the first operation on the basis that this action fulfilled the ‘Bolam test’ (i.e. a body of surgeons faced with the same clinical scenario would reasonably choose not to close the mesenteric defects as the evidence for its benefits at the time of surgery was equivocal). However, the experts were very critical of the failure of the bariatric surgeon not to diagnose an internal hernia of the biliopancreatic limb. Although the bariatric surgeon did perform an upper GI endoscopy and gastrograffin swallow, these investigations do not adequately delineate the anatomy of the biliopancreatic limb. In the context of a patient presenting with nausea and abdominal pain following gastric bypass, the failure to consider the diagnosis of internal herniation of the biliopancreatic limb and to arrange a CT scan or diagnostic laparoscopy to exclude this possibility was a breach of duty of care. In addition, the lack of clear written advice given to the primary physician by the surgeon following the patient’s discharge from the surgeon’s care was deemed to fall below the expected standard. Although the experts were more sympathetic toward the gastroenterologists, their overall opinion was that their failure to appreciate the severity of the patient’s symptoms, her malnourished status, and to either make a timely diagnosis of internal herniation of the biliopancreatic limb, or failing that, to urgently refer the patient on to a bariatric surgeon for an opinion about the cause of her malnutrition was a breach of duty of care. Overall, the collective negligence of the medical teams looking after the patient meant that she suffered from a potentially treatable pathology which directly led to her demise.

#### Learning Points

This case highlights the importance of initiating timely and appropriate investigations for postoperative bariatric patients. In addition, although bariatric patients are often discharged from the care of their primary surgeon, there is a responsibility on the surgeon to ensure that there is appropriate handover. Similarly, any team accepting responsibility for the management of bariatric patients’ needs to be competent in the management of post-bariatric complications, or at the very least have access to a specialist bariatric service to which they can refer for advice and support.

### Case Number 3

A 35-year-old female with a BMI of 47 kg/m^2^ and a past medical history of diabetes mellitus type 2 and hypertension was referred for bariatric surgery. Her case was discussed by the multidisciplinary team (MDT) at a regional bariatric surgical centre and she was listed for a laparoscopic Roux-en-Y gastric bypass (LRYGB). The procedure was uneventful and the patient was discharged on day 2. A fortnight after her surgery, the patient acutely presented to her local hospital with abdominal pain and vomiting. The patient was transferred to a bariatric centre after being managed in her local hospital over the weekend. Two days following transfer, she underwent a diagnostic laparoscopy which revealed peritonitis over all the four quadrants of the abdomen. Her procedure was converted to laparotomy and during dissection of the gastro-jejunostomy, a splenic laceration was noted. A splenectomy was performed, the peritoneal cavity washed out and intra-abdominal drains were inserted. Following a prolonged period in the intensive care unit the patient was transferred to the ward and subsequently to the community rehabilitation service.

#### Medicolegal Analysis

The patient’s solicitors originally submitted a letter of claim alleging the following:

i. The patient was inappropriately discharged following her LRYGB.
ii. Following admission to her local hospital, there was an inappropriate delay in the transfer to the bariatric centre.
iii. The fact that the patient had a splenectomy was evidence of substandard performance of repeat surgery.

#### Expert Review

The experts felt that the initial operation was satisfactory as was the decision to discharge the patient. With respect to the issue of transferring back to the bariatric centre, there was good documentary evidence that the bariatric centre was called following admission and appropriate advice was given and enacted upon. In the experts’ opinion, urgent transfer to the bariatric centre would not have led to a change in management. Similarly, the experts felt that the decision to perform a laparoscopy with conversion to laparotomy was reasonable and the splenic injury, while unfortunate, was not evidence of negligence. However, on closer examination of the complete medical records, it was noted that the patient had presented to her general practitioner (GP) seven days following discharge with tachycardia and pyrexia. From the clinical records, it appeared that her primary physician was under the impression that the patient had undergone a gastric band insertion and treated her conservatively. The patient then represented 10 days after the surgery to the GP with pyrexia and abdominal pain. The patient was reassured by the GP who did not contact the bariatric team or the on-call surgeons at the local hospital. This failure to appreciate the severity of the patient’s symptoms was deemed to be a breach of duty of care and the delay in diagnosing the leak was deemed to be significant. On the balance of probabilities, an earlier diagnosis would have lessened the severity of the sepsis and peritonitis allowing for an enhanced recovery.

#### Learning Points

This case demonstrates the importance of the non-specialist in the management of bariatric complications. In particular, while the GP would not necessarily have been expected to diagnose the patient’s leak, his failure to contact the bariatric centre for advice was deemed to be a materially significant breach of duty of care.

### Case Number 4

A 45-year-old female with a BMI 60 kg/m^2^ was referred by her GP to her local NHS bariatric unit. She attended her initial assessment there and was informed about the long waiting times for publicly funded surgery. She then consulted her GP explaining that the prolonged wait for surgery was impacting her physical and mental health and therefore she was referred to a private bariatric surgeon for consideration of surgery. During this consultation, the patient disclosed that she was recently an inpatient in a psychiatric unit for severe depression and that the psychiatric unit was supportive of the patient’s decision to seek bariatric surgery. The patient was referred on for further assessment by the counsellor and dietitian who both felt that she was an appropriate candidate for bariatric surgery. The patient went on to have LSG, which was uneventful. However, on the postoperative day 5, the patient represented to hospital with severe abdominal pain. She underwent a diagnostic laparoscopy where a staple line leak was identified in the proximal stomach. This was managed with the insertion of drains and endoscopic stenting. The patient required a prolonged hospital stay following which she had a significant deterioration in her mental health status requiring psychiatric input.

#### Medicolegal Analysis

The patient’s solicitors submitted a letter of claim stating the following:

i. The psychological assessment carried out preoperatively for the patient by the counsellor was inadequate and the patient did not receive an assessment from a consultant psychiatrist.
ii. A reasonable MDT would not have proposed bariatric surgery for a patient so soon after requiring admission to a psychiatric unit. There was also a lack of a minuted MDT meeting discussion.
iii. Had the surgical procedure been delayed, she would have been in a more robust state of mind to tolerate any possible complication and therefore not have suffered her mental health sequelae.

#### Expert Review

The experts felt that since the patient did undergo an assessment by a surgeon, dietician, and counsellor, in the context of a private service, the absence of a minuted formal MDT meeting was not a breach of duty of care. The experts were, however, critical of the failure of the surgeon to critically evaluate the patient’s self-reported statements regarding her mental health status. It was felt that in the context of her recent admission under the psychiatrist, the failure of the surgeon or counsellor to request details from her psychiatrist was a breach of duty of care. However, with regard to the significance of this breach, it was noted that the patient had requested bariatric surgery, had a very high BMI, and had been referred by her GP (who was aware of her mental health status). An expert psychiatrist was instructed who concluded that while mental health deterioration was a potential consequence following bariatric surgery, there was no specific intervention which could have been undertaken preoperatively to have reduced this risk. Therefore, the experts concluded that though there was a breach of duty of care in failing to contact the patient’s psychiatrist, it was clear that the patient wished to proceed with the surgery. They added that if all the information had been made available to the MDT (including a summary of her psychiatric history), the outcome of the MDT would have been that on the balance of probabilities, the risks of surgery were outweighed by its potential benefits and therefore the MDT would not have approved the patient for surgery. Hence, on the basis of a failure to prove causation, the patient’s claim was not successful.

#### Learning Points

This case illustrates the importance of a comprehensive preoperative MDT assessment prior to proceeding with bariatric surgery. Patients undergoing surgery are often psychologically vulnerable and may ascribe any mental health issues to their obesity. Such patients may therefore be keen to proceed with bariatric surgery and it is important to seek independent objective reviews of their medical, psychological, surgical, and dietetic status in order to make an informed MDT decision as to the advisability of surgery.

## Conclusion

Bariatric surgery requires a unique paradigm with a multidisciplinary approach both pre- and postoperatively to improve the long-term functional outcomes of patients. The outcome of bariatric surgery is improving in the UK, continental Europe and the USA; however, there is an increase in the overall number of cases leading to higher number of litigations which are not confined to the performance of the operations alone. With an appropriate consent process, careful surgical technique, multidisciplinary involvement pre- and postoperatively and robust follow-up protocols the risks can be reduced and mitigated. Specific issues relate to consent, lack of follow-up, delayed identification of complications, as well as poor emergency management of complications. These can be directed towards various specialties including bariatric surgeons, clinicians, GPs and MDT members. A Medicolegal checklist with the most common factors influencing the frequency and outcomes of litigations is presented in Table 2. In countries, such as the UK or Canada, with predominantly state-funded health care systems, there is a growing issue with access to publicly funded bariatric surgery and it is likely that rationing of bariatric surgery driving patients to undergo weight loss surgery abroad. Most surgeons in the UK are experiencing an influx of patients presenting with severe complications of bariatric surgery undertaken outside the UK. The concept of medical tourism is not new or specific to the UK but was mostly undertaken for the purpose of dental and aesthetic surgery in the past [33, 34]. However, bariatric surgery is more complex requiring careful patient selection, an MDT approach and close follow-up. Furthermore, complications in bariatric surgery can sometimes be devastating and difficult to deal with [35–38]. It is likely that these cases will become a significant source of medicolegal claims in the future.

**Table 2:** Medicolegal checklist of influencing factors of litigation

**General**	Adhere to guidelines provided in all aspects of the bariatric surgical care, for example indication, investigation, surgical technique, by relevant organisations such as ASMBS, IFSO or NICE. In addition, ensure all patients have been discussed at a specialist bariatric multidisciplinary team meeting
	Ensure that all documentation, including medical records, surgical notes and consent forms are clear, legitimate and accurately reflects the consultation that was held with the patient
	Communicate clearly and use adjuncts such as leaflets, images and videos. Provide ample opportunity for the patient to ask questions and clarify any doubts. Offer patients with contact information of the Bariatric Team in order to can query waiting times and book appointments
**Consent**	Obtain informed consent from patients in advance of the day of the operation. Explain the surgical and conservative options available, explain the different type of operations and outcomes as well as the risks and benefits associated with each of them
	Consent form: ensure that all possible surgical and conservative short- and long-term complications risks are presented to the patient including changes to body shape, psychological issues and weight regain
	Avoid consenting on the day of the operation but do confirm the consent
**Technical factors**	Conduct appropriate preoperative investigations, such as blood tests, imaging studies and endoscopy, to ensure that the patient is a suitable candidate for surgery. Ensure patient has been cleared by dietitians and clinical psychology
	Perform operation competently by using careful surgical technique with attention to details. Avoid all but particularly gross technical errors at all cost (such as Roux-en-O)
	Construct a clear operation note, include a clear description of the surgical technique and equipment used, such as staplers, size of the bougie and any other relevant details
	Conduct quality control of the operation (for example a leak test, record the surgery and take intraoperative photographs)
**Postoperative care**	Conduct daily reviews of the patient’s progress post-surgery and document the surgical plan each day, with particular consideration of pain and nausea management
	Avoid failure to rescue by effective communication with team members, regular monitoring of the patients and have an emergency response plan/investigation sequence in place
	Provide appropriate long term postoperative care, including monitoring for complications, providing nutritional guidance and adequate outpatient follow-up

*ASMBS* American Society for Metabolic and Bariatric Surgery, *IFSO* International Federation for the Surgery of Obesity and Metabolic Disorders, *NICE* National Institute for Health and Care Excellence

Bariatric surgeons must continue to evolve their practice accordingly and standardise their surgical techniques for each procedure in order to reduce errors and potential harm to patients (39). To ensure expertise and provide optimal outcomes, formal bariatric fellowship programmes are established to train surgeons in these techniques and maintain high standards of practice. In addition, many countries, including the UK, have developed surgical registries and professional societies to provide a more regulated service and to monitor and improve patient outcomes.

In conclusion, there is a rising incidence of medicolegal claims following bariatric surgery. The underlying reasons for this are multifactorial including an increase in the volume of surgery, high patient expectations, the incidence of long-term postoperative complications and the requirement of long-term follow-up. In order to avoid medicolegal pitfalls, it is important to ensure that the consenting process is comprehensive and that every complication is appropriately investigated and managed within a timely manner. It is likely that over time, there will be increasing litigations relating to the management of the patients presenting as an emergency with long-term bariatric complications and also due to patients who are placed on long waiting lists for bariatric surgery within the public-funded health systems.
